# Research progress and perspectives of N-methyl-D-aspartate receptor in myocardial and cerebral ischemia-reperfusion injury: A review

**DOI:** 10.1097/MD.0000000000035490

**Published:** 2023-10-20

**Authors:** Wei Liao, Yuehui Wen, Shaochun Yang, Yanyu Duan, Ziyou Liu

**Affiliations:** a Department of Neurosurgery, First Affiliated of Gannan Medical University, Ganzhou, Jiangxi, China; b Key Laboratory of Prevention and Treatment of Cardiovascular and Cerebrovascular Diseases, Ministry of Education, Gannan Medical University, Ganzhou, Jiangxi, China; c Shanghai Jiao Tong University School of Medicine, Shanghai, China; d Heart Medical Centre, First Affiliated of Gannan Medical University, Ganzhou, Jiangxi, China; e Department of Cardiac Surgery, First Affiliated of Gannan Medical University, Ganzhou, Jiangxi, China.

**Keywords:** cardiovascular ischemic diseases, cerebrovascular ischemic diseases, glutamate excitotoxicity, ischemia-reperfusion injury, NMDAR

## Abstract

There is an urgent need to find common targets for precision therapy, as there are no effective preventive therapeutic measures for combined clinical heart-brain organ protection and common pathways associated with glutamate receptors are involved in heart-brain injury, but current glutamate receptor-related clinical trials have failed. Ischemia-reperfusion injury (IRI) is a common pathological condition that occurs in multiple organs, including the heart and brain, and can lead to severe morbidity and mortality. N-methyl-D-aspartate receptor (NMDAR), a type of ionotropic glutamate receptor, plays a crucial role in the pathogenesis of IRI. NMDAR activity is mainly regulated by endogenous activators, agonists, antagonists, and voltage-gated channels, and activation leads to excessive calcium influx, oxidative stress, mitochondrial dysfunction, inflammation, apoptosis, and necrosis in ischemic cells. In this review, we summarize current research advances regarding the role of NMDAR in myocardial and cerebral IRI and discuss potential therapeutic strategies to modulate NMDAR signaling to prevent and treat IRI.

## 1. Introduction

Cardiovascular and cerebrovascular diseases have a high incidence and are associated with high rates of disability and mortality, imposing a heavy burden on families and society. Currently, effective preventive and therapeutic measures for cardio-cerebral damage caused by these diseases are lacking. Increasing evidence suggests that there may be a common injury mechanism between the heart and brain, and the concept of joint heart-brain protection and treatment for cardiovascular and cerebrovascular diseases has become the focus and trend of current research.^[1–3]^ The exploration of joint heart-brain protection and treatment is beneficial for the systematic study of ischemic diseases of the cardiovascular and cerebrovascular system.

Ischemia-reperfusion injury (IRI) is a common pathological condition that occurs in the heart and brain during the pathogenesis of ischemic diseases of the heart and brain and can lead to severe morbidity and mortality.^[4]^ IRI is characterized by a paradoxical exacerbation of tissue damage during the restoration of blood flow after a period of ischemia, which leads to oxidative stress, inflammation, apoptosis, and necrosis.^[5]^ IRI is involved in many clinical situations such as myocardial infarction, ischemic stroke, cardiac arrest, organ transplantation, and vascular surgery.^[6]^ Glutamate, as a major excitatory neurotransmitter in the central nervous system, plays a crucial role in the pathogenesis of IRI through the activation of different types of glutamate receptors, such as N-methyl-D-aspartate receptor (NMDAR), α-amino-3-hydroxy-5-methyl-4-isoxazolepropionic acid (AMPA), etc.^[7]^ NMDAR is widely distributed in organs such as the heart, brain, and kidney, and is normally involved in processes such as synaptic plasticity and regulation of myocardial function. After IRI, glutamate is released in large quantities, which further activates NMDAR and leads to an excessive influx of calcium ions into ischemic cells, oxidative stress, mitochondrial dysfunction, inflammation, apoptosis, and necrosis,^[8]^ which mediate a variety of physiologic and pathologic processes, such as synapse, transmission, learning and memory, neurogenesis, cardioprotection, and neuroprotection, etc. In recent years, pharmacological or non-pharmacological intervention strategies targeting NMDAR or its downstream signaling pathways have been widely investigated to attenuate or prevent IRI, providing new ideas and targets for the diagnosis and treatment of IRI.^[9,10]^

In this review, we first introduce the classification and structure of NMDAR and review the mechanism of NMDAR in myocardial and cerebral IRI, respectively. Then, we focus on the targets of NMDAR signaling as well as the therapeutic strategies and methods and present our views on future research directions. By summarizing the role of NMDAR in cardiac and cerebral IRI and the research progress, we aim to explore the joint protection of the heart and brain and the treatment of the heart and brain together.

## 2. Structure and distribution of NMDAR

**Figure 1.**
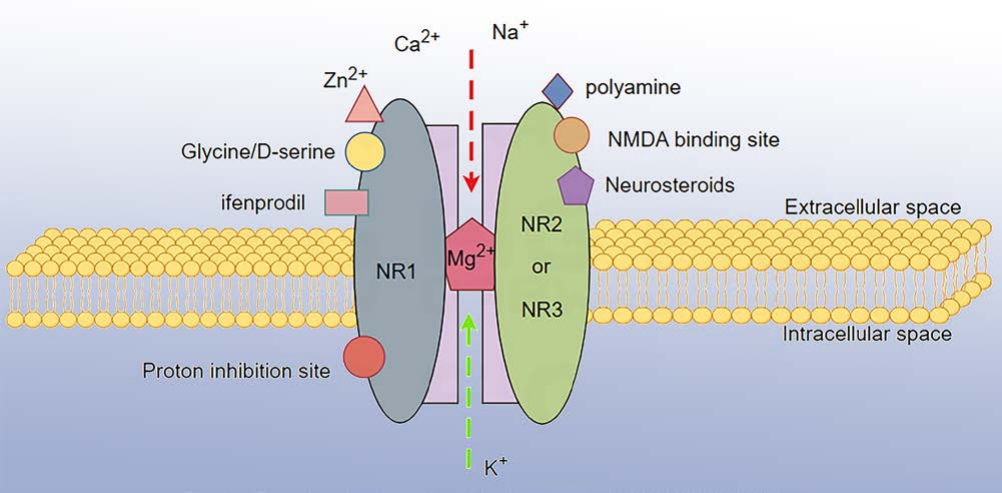
Structure and binding site of NMDAR. NMDAR is a heterotetrameric complex consisting of 2 NR1 subunits and 2 NR2 (A-D) or NR3 (A-B) subunits. NMDAR is a nonspecific cation channel. NMDAR activation leads to intracellular K^+^ efflux and extracellular Na^+^ and ca^2+^ efflux. NMDAR = N-methyl-D-aspartate receptor, NR1 = NMDA Receptor 1, NR2 = NMDA Receptor 2, NR3 = NMDA Receptor 3.

NMDAR is an ionotropic glutamate receptor, NMDAR is a heterotetrameric complex consisting of 2 obligate GluN1(NR1) subunits and 2 GluN2(NR2) (A-D) or GluN3(NR3) (A-B) subunits.^[11]^ All NMDA subunits share a common membrane topology, including an extracellular amino-terminal structural domain, an extracellular ligand binding domain formed by the S1 and S2 fragments, 4 membrane structural domains, and an intracellular C-terminal structural domain.^[12]^ The glycine binding sites in the ligand binding domains of the various GluN subunits play different functional roles in NMDAR. The regulation of NMDAR activity relies on 3 main aspects: endogenous regulators, voltage-gated and specific activators, and inhibitors.^[13,14]^ The structure and binding site of NMDAR are shown in Figure 1. In the past, most of the studies on NMDAR have focused on neurological disorders, and in recent years, with more and more in-depth studies, it has been shown that glutamate is also involved in the regulation of physiopathological functions of peripheral tissues, including the lungs, kidneys, liver, heart, gastric, and immune system,^[15,16]^ and also plays an important role in these non-neurological disorders. In renal IRI, the expression of NMDAR isoforms and subunits varies in different renal regions and cell types resulting in different sensitivities and severities of renal IRI, and blockade of NMDAR can attenuate renal IRI and protect renal function and structure.^[17]^ Especially in the cardiovascular system, studies have shown that NMDAR is detected and expressed throughout the cardiovascular system in both the physical and pulmonary circulation, such as the conduction system, cardiomyocytes, pachyderm fibers, sinus node, etc,^[18]^ and plays different roles.

## 3. Role of NMDAR in myocardial IRI

**Figure 2.**
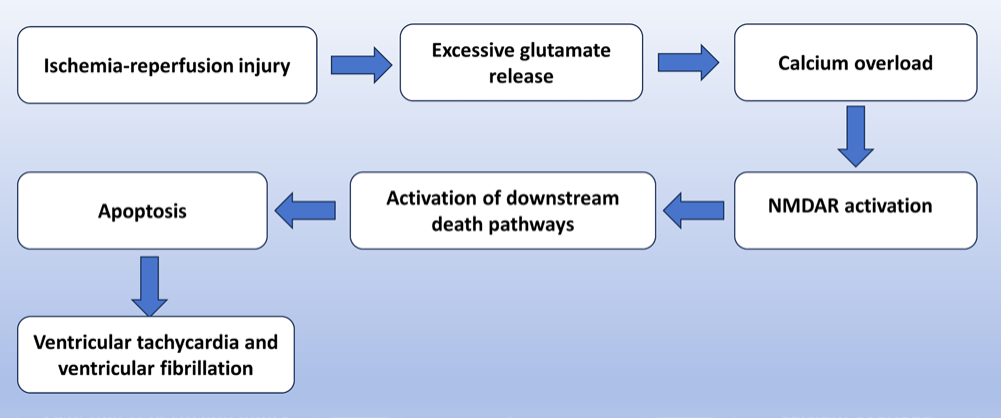
NMDAR cascade activated after myocardial IRI. Myocardial IRI leads to excessive glutamate release and intracellular calcium aggregation, which overloads the NMDAR, thereby activating the downstream death pathway leading to apoptosis of cardiomyocytes, and ultimately to ventricular tachycardia and ventricular fibrillation. IRI = ischemia-reperfusion injury, NMDAR = N-methyl-D-aspartate receptor.

NMDAR is extensively distributed in cardiac tissue and plays a crucial role in regulating a range of physiological and pathological mechanisms, including cardioprotection, preconditioning, postconditioning, arrhythmias, hypertrophy, and apoptosis.^[18–20]^ Myocardial ischemia is characterized by a deficiency in oxygen and glucose, which hinders the production of ATP and promotes anaerobic glycolysis. Consequently, this metabolic shift leads to acidosis and the accumulation of lactate. Insufficient blood supply to the coronary arteries leads to impaired oxidative phosphorylation in cardiomyocytes, resulting in reduced ATP production, decreased sodium pump activity, and a decrease in the intra- and extracellular sodium ion concentration gradient. At the same time, cardiomyocytes secrete significant quantities of glutamate into the extracellular space. When reperfusion takes place, molecular oxygen is introduced into the myocardial tissue through the blood, triggering the activation of the Na^+^/Ca^2+^ exchange protein. This protein facilitates the transportation of Na^+^ to the extracellular space in a reverse mode, leading to an elevation in intracellular Ca^2+^ levels. Consequently, this process induces cellular injury.^[21]^ In addition, the activation of glutamate receptors leads to an increase in intracellular calcium ions, which subsequently initiates a cascade of pathophysiological events, including ventricular fibrillation and ventricular tachycardia.^[22]^ The Figure 2 illustrates the activation of the NMDAR cascade response following myocardial IRI.

NMDAR is a voltage-dependent calcium channel receptor that is normally blocked by Mg^2+^. When glutamate binds to the receptor and induces depolarization of the cell membrane, the channel undergoes removal of Mg^2+^, thereby facilitating the entry of Ca^2+^ into the cell. NMDAR-mediated calcium (Ca^2+^) influx has the ability to initiate multiple signaling pathways, including the mitochondrial pathway, the endoplasmic reticulum pathway, the phospholipase C pathway, the nitric oxide synthase pathway, and the heat shock protein pathway. These pathways can ultimately result in apoptosis or necrosis. Activation of NMDAR can also lead to an elevation in the generation of reactive oxygen species, resulting in lipid peroxidation and impairment of the cell membrane. This, in turn, triggers inflammatory reactions, enhances the adhesion and infiltration of leukocytes, releases inflammatory mediators and cytokines, influences gene expression, and modulates cellular processes such as the cell cycle, apoptosis, and autophagy.^[18]^ The involvement of NMDAR in the pathogenesis of myocardial IRI has been substantiated through multiple investigations.^[23–25]^ Studies have demonstrated that the NMDAR antagonist MK-801 has the potential to decrease the extent of myocardial IRI, enhance cardiac function, and reduce the apoptosis rate and levels of free radicals in cardiomyocytes.^[20,26,27]^ Additionally, the NMDAR antagonist memantine has been found to exert cardioprotective effects in animal and cellular models of myocardial infarction, possibly through the attenuation of pro-inflammatory and oxidative stress factors, leading to a subsequent reduction in cardiac remodeling. In relation to the mitigation of pro-inflammatory and oxidative stress factors and consequent cardiac remodeling, memantine has the ability to activate the brain-derived neurotrophic factor (BDNF)/TrkB signaling pathway in HUVECs. This activation helps in reducing inflammation and oxidative stress induced by oxidized-LDL, and thus, memantine has been proposed as a potential cardioprotective agent.^[28–30]^ Sympathetic excitation in chronic heart failure is strongly associated with the NMDAR subunit NR1.^[31]^ Additionally, the inhibition or reduction of the NMDAR subunit NR1B has been shown to provide protection against myocardial IRI.^[32]^ The above studies fully demonstrate that NMDAR can play a role in myocardial IRI, which can lay the foundation for our next in-depth study of the mechanism of NMDAR in myocardial IRI, and at the same time, it can provide a direction for further exploring the common injury pathway of the heart and brain. The effects of NMDAR on myocardial IRI and the mechanism of NMDAR are shown in Table 1.

**Table 1:** Effects and mechanisms of NMDAR on myocardial IRI.

NMDAR/subunit	Species	Role in myocardial IRI	Mechanisms	Ref
NMDAR	Cells	Cardiomyocyte viability apoptosis.	High NMDAR-driven Calcium inward flow leads to apoptosis through the P38MAPK pathway.	^[26]^
NMDAR	Rat	Effects on cardiac remodeling function in vivo and in vitro.	NMDAR inhibitors result in reduced proinflammatory, oxidative stress factors and Cardiac Fibrosis and hypertrophy	^[28]^
NMDAR	Mice	Influence on apoptosis after cardiac IRI.	MiR-219a-2 attenuates apoptosis in IRI by reducing NMDAR-mediated calcium overload through the HIF1α/NR1 axis.	^[23]^
NMDAR-1	Mice/Cells	Decreased myocardial contractility.	Induces mitochondrial dysfunction in cardiomyocytes, leading to decreased myocardial contractility	^[32]^
NMDAR	Rat	Induction of tardive dyskinesia after myocardial IRI.	Reduction of calcium loading in mitochondria.	^[33]^
NMDAR/AMPAR	Rat	Increased occurrence of ventricular tachycardia and ventricular fibrillation.	Upregulation of ionotropic glutamate receptor expression by mediating platelet activation.	^[34]^
NMDAR	Rat	Caused by reperfusion injury arrhythmia, not ischemia.	Undetermined.	^[35]^
NMDAR	Rat	reduced left ventricular function, elevated expression of cardiac enzyme profiles.	Inhibits NMDAR activity, improves Ca^2+^-ATPase activity, and reduces intracellular Ca^2+^accumulation.	^[20]^
NMDAR	Cells	Significantly improves cardiac function and delays myocardial fibrosis.	Reduction of apoptosis in damaged cardiomyocytes and inhibition of cardiomyocyte apoptosis using NO-SMS formulation.	^[36]^
NMDAR	Rat	Involved in lactate elevation after myocardial ischemia.	Undetermined.	^[37]^
NMDAR	Rat	Improves cardiac function reduces oxidative stress	Undetermined.	^[38]^

For detailed information, see the text. AMPAR = α-amino-3-hydroxy-5-methyl-4-isoxazolepropionic acid receptor, ATPase = adenosine triphosphate Synthase, HIF-1α,hypoxia inducible factor-1α; IRI = ischemia-reperfusion injury, MAPK = mitogen-activated protein kinase, NMDAR = N-methyl-D-aspartate receptor, NO-SMS = new optimized Sheng-Mai-San, NR1 = N-methyl-D-aspartate receptor1, Ref = reference.

### 3.1. Mechanism of NMDAR in myocardial IRI

Combined with the literature, the mechanism of NMDAR-mediated myocardial injury is complex and involves a variety of signaling pathways and cellular processes. Combined with the current study, it has been shown that the mechanism of NMDAR in myocardial IRI mainly involves the following aspects:

Calcium overload and cytotoxicity: Activation of NMDAR increases intracellular calcium ion levels, leading to calcium overload and cytotoxicity. Calcium overload can activate a variety of calcium-dependent enzymes, such as phospholipases, protein kinases, and protein hydrolases, resulting in membrane phospholipid degradation, protein denaturation, increased production of oxygen free radicals, mitochondrial dysfunction, and decreased ATP production, which ultimately leads to apoptosis or necrosis.^[23,26]^

Free radical production: Activation of NMDAR increases intracellular calcium ion levels and promotes free radical production. Free radicals can cause lipid peroxidation and cell membrane damage, destroying the normal structure and function of the membrane, altering the normal function of blood vessels, and reducing ATP production. Free radicals can also react with proteins and nucleic acids, leading to protein function inhibition, chromosomal aberrations, and DNA breaks.^[22,30]^

The inflammatory response: Activation of NMDAR increases intracellular calcium ion levels and stimulates an inflammatory response. The inflammatory response can increase the adhesion and infiltration of leukocytes, and release inflammatory factors and cytokines, such as TNF-α, IL-1β, IL-6, etc, resulting in microvascular damage, increased permeability, no-reflow phenomenon, and tissue edema. Inflammatory factors and cytokines can also affect gene expression and regulate processes such as cell cycle, apoptosis, and autophagy. Gene expression: activation of glutamate receptors increases intracellular calcium ion levels and affects gene expression. Gene expression can regulate processes such as cell cycle, apoptosis, and autophagy, such as p53, B-cell lymphoma 2 (Bcl-2), Bax, and caspase. Gene expression can also affect myocardial remodeling and cardiac function recovery, such as MMPs, TIMPs, TGF-β, etc.^[28]^

### 3.2. Biomarkers of NMDAR activation in myocardial IRI

Biomarkers are measurable indicators of biological processes or conditions that can be used in the diagnosis, prognosis, or therapeutic evaluation of diseases. Biomarkers of NMDAR activation in myocardial IRI may indicate the level of glutamate release, uptake, or metabolism, as well as the subsequent impact of NMDAR signaling on cellular damage or repair. Biomarkers of NMDAR activation in myocardial IRI can be detected in various of biological samples including blood, urine, or tissue.

Another biomarker of NMDAR receptor activation in myocardial IRI is the expression level of glutamate receptor subunits or related proteins in ischemic myocardium, which reflects the regulation of glutamate receptor signaling or function through transcriptional or post-transcriptional mechanisms. The expression levels of glutamate receptor subunits or related proteins can be determined by various methods, such as immunohistochemistry, Western blotting, or quantitative polymerase chain reaction. For example, the expression levels of NMDA receptor subunits are elevated in the ischemic myocardium of rats with myocardial infarction.^[32]^

The third biomarker of glutamate receptor activation in myocardial IRI is the level of activity of downstream signaling pathways or cellular processes affected by glutamate receptor signaling. These processes include calcium influx, oxidative stress, mitochondrial dysfunction, inflammation, apoptosis, or necrosis. The level of activity of downstream signaling pathways or cellular processes can be measured by various methods, including calcium imaging, reactive oxygen species (ROS) assays, mitochondrial membrane potential measurements, cytokine assays, caspase activity assays, or lactate dehydrogenase release assays.^[26,36,37]^

## 4. Role of NMDAR in cerebral IRI

**Figure 3.**
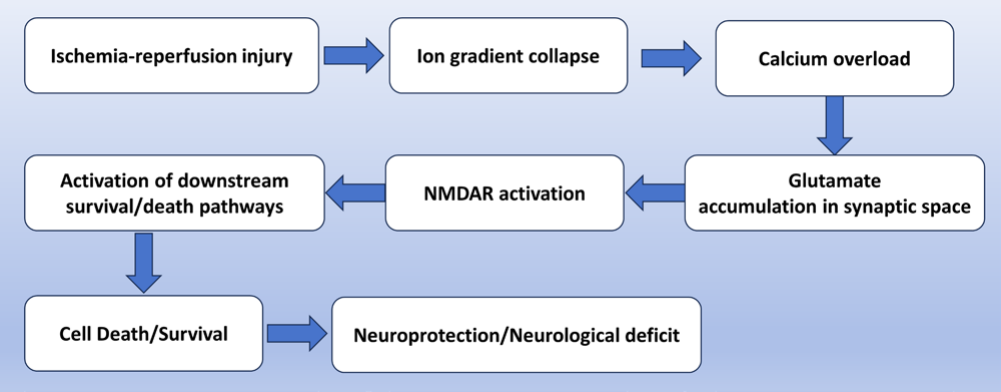
NMDAR cascade activated after cerebral IRI. Cerebral IRI leads to a collapse of the ionic gradient and an overload of intracellular calcium ions, which results in a massive release of glutamate and activation of NMDAR, ultimately leading to the activation of downstream death and survival pathways, which can either protect or cause neurological deficits. IRI = ischemia-reperfusion injury, NMDAR = N-methyl-D-aspartate receptor.

Excitotoxicity induced by glutamate signaling is one of the pathogenic mechanisms of neuronal cell death, which can occur in various neurological disorders such as ischemic-hypoxic stroke, epileptic brain injury, Alzheimer disease, and other neurological disorders.^[39,40]^ Under physiological conditions, glutamate supports neuronal survival and synaptic plasticity. However, under pathogenic conditions, the excessive release of extracellular glutamate can stimulate glutamate receptors, inducing calcium in-flow and ROS accumulation, leading to the activation of calcium-dependent death signaling pathways. The toxicity of glutamate in cells and tissues after resuscitation is not immediately alleviated and can even worsen the injury.^[18,41,42]^ NMDAR is widely expressed in the brain, mediating various physiological and pathological processes, such as synaptic transmission, learning and memory, neurogenesis, neuroprotection, and neurodegeneration.^[43,44]^ During cerebral ischemia, hypoxia, and glucose deprivation lead to impaired ATP production and increased anaerobic glycolysis, resulting in acidosis and accumulation of lactate. These metabolic changes lead to the release of glutamate from neurons and astrocytes through the reverse operation of sodium-dependent glutamate transporters (GLAST, GLT-1, EAAC1, EAAT4, and EAAT5). Upon reperfusion, blood flow is restored, which flushes out accumulated metabolites and generates ROS. recovery leads to further glutamate release and NMDAR overactivation, generating a series of cascade responses and activating downstream cell survival and death pathways that act as neuroprotective or injurious,^[40]^ and the cascade of responses resulting from activation of NMDAR after cerebral IRI is shown in Figure 3.

**Figure 4.**
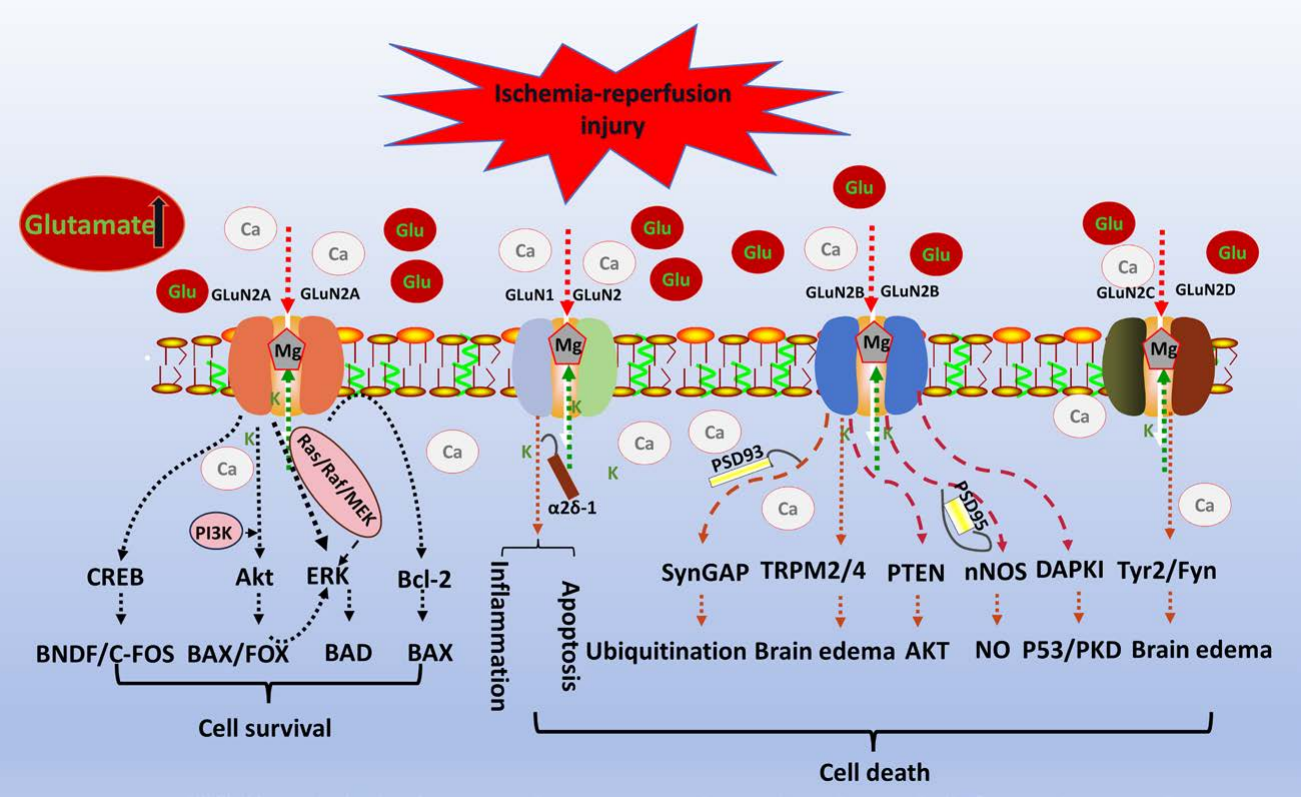
Cell survival and death mechanisms induced by different subunits of NMDAR after cerebral IRI. After the onset of IRI, glutamate excitotoxicity is enhanced, leading to the inward flow of extracellular Ca^2+^, the outward flow of K^+^_,_ and the removal of Mg^2+^ from the channel, which in turn triggers a series of cascade reactions, among which GLuN2A mainly plays a role in protecting the cells and promoting the survival after the IRI, whereas GLuN1 with GLuN2B/C/D mainly acts as a catalyst for cell death. Akt: Protein kinase B, BAD = Proteins of the B-cell lymphoma-2 associated death promoter, BAX = Proteins of the B-cell lymphoma-2 associated X, Bcl-2 = B-cell lymphoma-2, BDNF = brain-derived neurotrophic factor, C-FOS = Proto-oncogene FOS, CREB = cAMP response element-binding protein, DAPK1 = death-associated protein kinase 1, ERK = extracellular regulated protein kinases, FOX = Forkhead box, Fyn = Src family tyrosine kinase, Glu = Glutamate, GLuN2A = NMDA Receptor 2A, GLuN2B = NMDA Receptor 2B, GLuN2C = NMDA Receptor 2C, GLuN2D = NMDA Receptor 2D, GLuN1 = NMDA Receptor 1, GLuN2 = NMDA Receptor 2, MEK = MAP kinse-ERK kinase, NMDAR = N-methyl-D-aspartate receptor, nNOS = nitric oxide synthase, PI3K = phosphoinositide 3–kinase, PKD = protein kinase D, PSD95 = postsynaptic density 95, PSD93 = postsynaptic density 93, PTEN = phosphatase and tensin homolog, Raf = Raf oncogene, Ras = resistance to audiogenic seizures, SynGAP = Synaptic Ras GTPase activating protein, TRPM2/4 = transient receptor potential melastatin 2/4, Tyr2 = tyrosine 2.

Current studies on NMDAR in cerebral IRI have primarily focused on the concept of glutamate excitotoxicity. Consequently, the neuroprotective approaches have been limited to blocking the overactivation of ionotropic glutamate receptors. However, disappointingly, due to the synaptic or extrasynaptic release process of glutamate, as well as the mechanism of action of different NMDAR subunits, the timing and duration of activation, the location and cell type of expression and interactions with other signaling pathways, as well as the reuptake of glutamate in the brain and blood after glutamate metabolism, the current clinical treatments designed to target the NMDAR after cerebral IRI often ended with a failure, too further explore the enhancement of the role of NMDAR in cerebral IRI and subsequent clinical applications, the “NMDAR subtype,” “NMDAR localization” and “NMDAR time “ 3 hypotheses are currently being discussed to describe the potential mechanisms of NMDAR in cerebral IRI^[45]^ and the mechanisms of the role of different NMDAR subunits in cerebral ischemia are shown in Figure 4. Therefore, the search for and development of new targets for neuroprotective interventions in the prevention and treatment of cerebral IRI remains the focus and goal of research in this field. Table 2 summarizes the main effects and mechanisms of NMDAR on cerebral IRI.

**Table 2:** Main effects and mechanisms of NMDAR on cerebral IRI.

NMDAR/subunit	Species	Role in cerebral IRI	Mechanisms	Ref
NMDAR	Mice	Neuronal death.	TRPM2-NMDAR association promotes the surface expression of extrasynaptic NMDAR, leading to enhanced NMDAR activity and increased neuronal death.	^[46]^
GLuN2A	Cells	Neuroprotection	Co-treatment with gardenia glycosides and GluN2A antagonists was able to exert a neuroprotective effect, whereas with GluN2B inhibitors failed to produce an effect.	^[47]^
GLuN2A	Cells	Blocking microglia-neuron interactions.	GluN2A but not GluN2B inhibitors block microglia-neuron physical interactions.	^[48]^
GLuN1/GLuN2A/GLuN2B	Cells/Rat	Reduced synapse-associated proteins and Ca^2+^	Amelioration of synaptic damage after cerebral ischemia/reperfusion injury by inhibition of NMDA receptor hyperactivation.	^[49]^
GluN2A/B	Mice	GluN2A excitotoxicity was enhanced and GluN2B excitotoxicity was attenuated.	Targeted exon exchange replaces the CTD of GluN2B with that of GluN2A reduces the vulnerability of forebrain neurons to NMDAR-dependent Ca^2+^ inward flow in vitro and in vivo, and the isoform of the C-terminal structure determines the magnitude of excitability.	^[50]^
GLuN2B	Mice	Neuronal cell death.	Ischemic neuronal death requires CaMKII binding to synaptic GluN2B, whereas any potential role for DAPK1 binding is limited to a distinct, possibly extrasynaptic GluN2B population	^[51]^
GLuN2B	Cells/Rat	Increased glutamate excitability and Ca^2+^ overload.	Cerebral ischemia-reperfusion injury directly and indirectly activates GluN2B and involves ubiquitination of GluN1B and MALT	^[52]^
GLuN2B	Cells	Attenuated hypoxia-induced oxidative damage in hippocampal neurons.	Inhibition of GluN2B-mediated excitotoxicity and suppression of ROS production.	^[53]^
GLuN2B	Mice	Prevents stroke damage and improves behavioral performance.	The GluN2B mutation inhibited extrasynaptic NMDA receptor currents without affecting synaptic NMDA receptor channel activity.	^[54]^
GLuN2B	Cells	Increased excitotoxicity.	Endocytosis of GluN2B-containing NMDA receptors mediates NMDA-induced excitotoxicity leading to neuronal apoptosis	^[55]^
GluN2C	Mice	Reduced cerebral edema and higher rates of neurologic recovery.	The GluN2C subunit promotes neuronal dysfunction in the penumbra region through reduced Fyn kinase expression at Tyr2 and reduced subunit phosphorylation.	^[56]^
GluN2C	Mice	Reduced calcium inward flow.	GluN2C was upregulated to promote neuronal survival after ischemia, and GluN2C knockout mice exhibited increased neuronal death and reduced spatial working memory in the CA1 region of the hippocampus.	^[57]^
GluN2C/D	Rat/Mice	Loss of white matter action potentials and severe functional deficits.	Release of axonal vesicular glutamate into the periaxonal space under the myelin sheath triggers activation of the myelin NMDA receptor containing the GluN2C/D subunit.	^[58]^
GluN2D	Mice	Increased excitotoxicity and neuronal death.	Increased cardiolipin synthesis and elevated sensitivity to apoptotic signaling.	^[59]^
GluN2D	Cells/Mice	Increased excitotoxicity.	tPA exerts deleterious effects on neurons through its action on GluN2D-containing neurons.	^[60]^

For detailed information, see the text. CaMKII = Calcium/calmodulin-dependent protein kinase II, DAPK1 = death-associated protein kinase 1, GLuN2A = NMDA Receptor 2A, GLuN2B = NMDA Receptor 2B, GLuN2C = NMDA Receptor 2C, GLuN2D = NMDA Receptor 2D, GLuN1 = NMDA Receptor 1, GLuN2 = NMDA Receptor 2, MALT = DNA-binding transcriptional activator MalT, NMDAR = N-methyl-D-aspartate receptor, ROS = reactive oxygen species, Tyr2 = tyrosine 2, tPA = tissue plasminogen activator.

### 4.1. NMDAR-mediated downstream survival pathways

NMDAR-mediated downstream survival pathways mainly include the cAMP response element-binding protein (CREB) signaling pathway, the protein kinase B (Akt) signaling pathway, the extracellular signal-regulated kinase (ERK) signaling pathway, and Bcl-2 family proteins.^[61]^ These signaling pathways can protect neurons from cerebral IRI by promoting neuronal growth, differentiation, survival, and synaptic plasticity. These signaling pathways are primarily activated by the NR2A subunit-containing NMDAR on the postsynaptic membrane. Additionally, it is now understood that after cerebral IRI, estrogens and other substances can have a neuroprotective effect through nonsynaptic NR2B-containing NMDA receptors.^[62]^

The CREB signaling pathway is an important transcription factor that regulates the expression of a variety of genes related to neuronal survival and function, such as BDNF, bcl-2, c-fos, etc. Activation of CREB requires the calcium and calmodulin-dependent protein kinase IV involvement, which in turn depends on the activation of NMDAR, which mediates survival channels that are transcription-dependent, and the transcription factor involved in this process may be cyclic adenosine monophosphate response element-binding (CREB) protein.^[63,64]^ Phosphorylated CREB after cerebral IRI can respond to gene transcription after reacting with binding proteins, and NMDAR can activate the release of downstream transcription factors to further promote CREB activation to play a role in protecting neurons, and during cerebral ischemia, Stimulation of extrasynaptic NMDAR induces CREB to shut down the pathway, leading to neuronal death.^[65]^

The Akt signaling pathway is an important cell survival factor that can resist a variety of cell death stimuli, such as hypoxia, free radicals, apoptosis-inducing factors, etc. The activation of Akt requires the involvement of phosphatidylinositol 3-kinase (PI3K) and phosphorylated inositol 3-kinase, and activated Akt is translocated to the nucleus, a process that promotes cell survival, whereas PI3K and phosphorylated inositol 3-kinase activation is dependent on NMDAR activation.^[66,67]^ Consistent with the survival-promoting effect of synaptic NMDAR, basal PI3K/Akt survival signaling is dependent on sustained synaptic activity and synaptic NMDAR activation. In addition, NMDAR can also directly activate Akt through a non-canonical mechanism that involves the activation of calcium-calmodulin-dependent protein kinase in a PI3K-dependent manner.^[68,69]^ Meanwhile, many previous studies have shown that inhibited Akt activation can lead to cerebral ischemia-induced neuronal death, whereas activation of Akt expression increases neural recovery after cerebral IRI.^[70–72]^

The ERK signaling pathway is a crucial factor in cell proliferation and differentiation. It regulates the expression of various genes related to neuronal growth and synaptic plasticity, including c-fos, zif268, Arc, and others. The activation of ERK relies on upstream molecules like Ras/Raf/MEK, which, in turn, depends on the activation of NMDAR.^[61,73]^

Bcl-2 family proteins are a class of important molecules that regulate apoptosis, some of which have anti-apoptotic effects, such as bcl-2, bcl-xL, etc, and some have pro-apoptotic effects, such as bax, bak, etc. Bcl-2 family proteins can regulate apoptotic cascade by affecting the mitochondrial membrane potential and cytochrome C release, etc. NMDAR can protect neurons against cerebral IRI by affecting the expression or function of the bcl-2 family proteins.^[72]^ Rho kinase inhibitors exert neuroprotective effects against cerebral ischemia, and their main mechanism is to reverse learning and memory deficits in a rat model of chronic cerebral ischemia by altering Bcl-2/Bax-NMDAR signaling in the cerebral cortex.^[74]^

### 4.2. NMDAR-mediated downstream death pathways

NMDAR-mediated downstream death pathways include calcium overload, free radical production, mitochondrial dysfunction, apoptotic cascade response, and inflammatory response. Often, the initiation of these downstream death pathways also requires the induction of neuronal toxicity through the activation of neuronal death-signaling complexes. These complexes include the GluN2B-PSD95-nNOS complexes,^[75,76]^ GluN2B-DAPK-P53 and GluN1-PTEN complexes, NMDAR-PSD93-SynGAP complexes, and NMDAR-TRPM complexes.^[45,72]^ These complexes further activate the downstream death pathways, ultimately leading to neurological deficits.

#### 4.2.1. α2δ-1-NMDAR complexes.

α2δ-1, a binding protein for gabapentin analogs encoded by the Cacna2d1 gene and thought to be a subunit of voltage-gated calcium channels, is a recently identified protein that can interact with NMDAR and is often currently used in the treatment of chronic neuropathic pain and epilepsy.^[77]^ It has been found that α2δ-1 can naturally form protein complexes with the C-terminal structure of NMDAR.^[78]^ Up to 40% of α2δ-1-bound NMDAR complexes are present on striatal cell membranes.^[79]^ Knockdown of the Cacna2d1 gene was similarly found not to increase basal NMDAR currents in an animal model of cerebral ischemia, suggesting that α2δ-1 may be required for ischemia-induced neuronal NMDAR pleiotropy in brain tissues, that concomitant ischemia can increase the association of α2δ-1 with NMDAR, and that formation of a complex by α2δ-1-bound NMDARs can mediate the cerebral ischemia/reperfusion injury after the process of neuronal cell death.^[80,81]^

#### 4.2.2. NMDAR-TRPM2/4 complexes.

Transient receptor potential cation channel, subfamily M (TRPM), transient receptor potential cation channel, after the occurrence of cerebral ischemia-hypoxia-reperfusion injury, TRPM2 is affected by oxidative stress and calcium-permeable channels and can influence NMDAR-induced excitotoxicity, while TRPM2 also forms complexes with NMDAR to further exacerbate the excitotoxic effects,^[46]^ TRPM4 in cerebral IRI similarly found that excitotoxic effects after cerebral IRI were caused by the physical coupling of TRPM4 and NMDAR, and that TRPM4 mainly interacted with GLuN2A and GLuN2B, but not other subunits, and that these complexes were mainly formed in synapses, and that TRPM2 was mainly formed in synapses of GLuN2A and GLuN2B, and no other subunits. These complexes are mainly formed outside the synapse, which partly explains why NMDAR exists to promote cell survival and cause cell death.^[82]^

#### 4.2.3. Fyn-PSD-93-GLuN2B complexes.

Postsynaptic density protein-93 (PSD-93) can bind to the C-terminal structure of NMDAR and is essential for NMDAR-mediated transactivation of cellular activity. PSD-53 deficiency inhibits neurotoxicity in cortical neurons activated by platelets.^[83]^ Additionally, PSD-93 deficiency in vivo and in vitro prevents ischemic injury. This effect may be achieved by PSD-93 acting as an anchoring protein to recruit Fyn and form complexes.^[84]^ Another recent study similarly found that PSD-93 directly interacts with SynGAP and mediates its ubiquitination and degradation, thereby exacerbating ischemic brain injury.^[85]^

#### 4.2.4. GLuN2B-PTEN complex.

Phosphatase and tensin homolog deleted on chromosome TEN (PTEN) is a phosphatase-tensin gene; in a cellular model of hypoxia-reoxygenation, it was found that it could interact with and form a complex with GluN2B, but not with GluN2A, and that knockdown of PTEN had a protective effect on hypoxia-reoxygenation-induced hippocampal neurons. Knockdown of PTEN has a protective effect on hypoxia-reoxygenation-induced hippocampal neurons, and its mechanism of action may be achieved by negatively regulating AKT signaling.^[86]^ In addition, the excitotoxic stimulation of NMDA can cause the translocation of PTEN nuclei in neurons, and it was found that the application of Tat-K13 interfering peptide not only reduced the ischemia-induced translocation of PTEN nuclei but also effectively reduced the ischemic brain injury at 6 hours after stroke in a focal ischemic rat model.^[87]^

#### 4.2.5. GLuN2B-PSD95-nNOS complexes.

The ability of excitotoxicity to activate postsynaptic density protein-95 (PSD-95) with neuronal NO synthase (nNOS) has been demonstrated^[75]^ as a downstream effector of excitotoxic cell death following IRI. After NMDA-induced elevation of calcium ions, PSD-95 utilizes its unique molecular structure to recruit calcium-dependent nNOS. This recruitment causes nNOS to translocate from the cytoplasmic matrix to the cell membrane, where it mediates the production of the neurotoxic molecule NO. The production of NO triggers a variety of downstream cell death events.^[88]^ On the other hand, when the structural association between PSD95 and nNOS is disrupted, it protects neurons and promotes neural recovery.^[76]^

#### 4.2.6. GLuN2B-DAPKI complexes.

Death-associated protein kinase 1 (DAPK1) is a pro-apoptotic kinase that is highly sensitive to the concentration of calcium ions, and it has been shown that it can bind to the C-terminal tail of GLuN2B to form a complex leading to a series of cell death processes such as excitotoxicity, and DNA breaks,^[89]^ Cerebral ischemia After IRI, excitotoxicity is able to activate postsynaptic density protein-95 PSD-95 with nNOS (neuronal NO synthase neuronal NO synthase) has been demonstrated,^[75]^ which are the downstream effectors of excitotoxic cell death, and after the occurrence of IRI, the After NMDA-induced elevation of calcium ions, PSD-95 utilizes its unique molecular structure to recruit calcium-dependent nNOS to translocate from the cytoplasmic matrix to the cell membrane and mediate the production of the neurotoxic molecule NO, which triggers a variety of downstream cell death events,^[88]^ whereas disruption of the structural association between PSD95 and nNOS protects neurons and promotes neural recovery.^[76]^

After reperfusion injury occurs, GLuN2B can form a complex with DAPKI, which increases the voltage of GLuN2B and increases cellular excitotoxicity, whereas exposure to the coupling of the 2 protects neurons,^[90]^ and it was similarly found in ischemic neuronal cells after cardiopulmonary resuscitation, where DAPK1 was able to bind with GLuN2B to promote neuronal cell death,^[51]^ and of course, after cerebral IRI Whether NMDA-induced cell death is caused by the GLuN2B-DAPKI complexes or by the terminal structure of GLuN2B alone is debatable, and it has been suggested that death-promoting NMDAR signaling is facilitated by the GluN1B C-terminus independent of DAPK1.^[91]^

### 4.3. Pharmacological intervention strategies targeting NMDAR or its downstream signaling pathways

Pharmacological intervention strategies that target the NMDAR or its downstream signaling pathways are potential therapeutic approaches for cerebral IRI. These strategies aim to protect neurons from cerebral IRI by inhibiting the over-activation of NMDAR or reducing NMDAR-mediated cellular damage. Several drugs or substances have been found to protect neurons from cerebral IRI by affecting the structure or function of NMDAR. These drugs can be categorized into the following main categories^[92]^:

NMDAR antagonists^[15]^: These drugs can directly or indirectly block NMDAR activity, thereby reducing calcium influx and excitotoxicity. For example, MK-801, ketamine, and memantine are a class of noncompetitive NMDAR antagonists that bind to the ion channels of the NMDAR and block cation passage; memantine and dextromethorphan are a class of low-affinity noncompetitive NMDAR antagonists that bind to the ion channels of the NMDAR but are effective only in the presence of high levels of glutamate; ifenprodil, Ro25-6981, etc are a class of selective NR2B subunit antagonists, which can bind to the NR2B subunit and inhibit NMDAR in the presence of high levels of NR2B,^[92]^ and AP5, AP7, etc are a class of competitive NMDAR antagonists, which can compete for the glutamate-binding site and prevent glutamate from activating NMDAR.^[45]^

NMDAR modulators: These drugs can improve cerebral IRI by affecting the expression, transport, assembly, and degradation of NMDAR to regulate the amount or function of NMDAR. For example, thioredoxin can ameliorate cerebral IRI by decreasing the expression of NR1 and NR2A subunits,^[93]^ thiol-based redox-active proteins can be cardioprotective therapeutic agents for cardiovascular diseases,^[94]^ and hydrogen sulfide can protect neurons by inhibiting phosphorylation of NR2B subunits and activation of CaMKII.^[95]^

Interventional agents of NMDAR downstream signaling pathways: These drugs can protect neurons from cerebral IRI by affecting a series of signaling molecules or pathways triggered by NMDAR activation, thus indirectly inhibiting NMDAR-mediated cellular injury. For example, free radical scavengers (e.g., vitamin E, vitamin C, glutathione, etc) can attenuate oxidative stress by scavenging free radicals^[96]^; calcium antagonists (e.g., nimodipine, verapamil, etc) can reduce calcium overload by blocking calcium channels^[97]^; and calcium-dependent enzyme inhibitors (e.g., a calpain inhibitor, caspase inhibitor, etc) can mitigate cellular damage by inhibiting calcium-dependent enzyme activity to attenuate cellular structural and functional damage^[98]^; neurotrophic factors (e.g., BDNF, nerve growth factor, etc) can enhance neuronal resistance by promoting neuronal growth and differentiation^[99]^; and anti-inflammatory drugs (e.g., nonsteroidal anti-inflammatory drugs, glucocorticosteroids, etc) can attenuate cellular damage by inhibiting inflammatory responses.^[100]^

### 4.4. Non-pharmacological intervention strategies targeting NMDAR or its downstream signaling pathways

In addition to pharmacological intervention strategies, several non-pharmacological interventions can attenuate or prevent cerebral IRI by modulating the NMDAR or its downstream signaling pathways, including the following main categories:

Physical interventions: This category refers to methods that protect neurons from cerebral IRI by altering the external environment or applying external stimuli to affect the activity of the NMDAR or its downstream signaling pathways. For example, hypothermia can reduce calcium overload and free radical production by decreasing the expression and phosphorylation of the NR1 and NR2B subunits,^[101]^ and hypoxic preconditioning can increase neuronal tolerance to IRI by increasing the NR2A/NR2B ratio^[102]^; Electrical stimulation can promote neuronal survival and functional recovery by increasing NR2A and decreasing NR2B expression.^[103]^

Biological interventions: This category refers to methods that protect neurons from cerebral IRI by modifying biological factors or applying biological stimuli to influence the activity of the NMDAR or its downstream signaling pathways. Gene therapy, for example, is an approach that utilizes genetic engineering techniques to repair or replace abnormal or missing genes. Gene therapy can regulate the function and expression of NMDAR by transfecting genes, such as antagonists or modulators of NMDAR subunits. This process helps protect neurons from cerebral ischemia/reperfusion injury.^[104,105]^ Stem cell therapy is a method of repairing or replacing damaged cells or tissues using stem cells that have the ability to self-renew and differentiate. Stem cell therapy can protect neurons from cerebral IRI by transplanting various types of stem cells, such as neural stem cells, mesenchymal stem cells, and embryonic stem cells, to promote neuronal regeneration and enhance functional recovery.^[106]^

Behavioral interventions: This category refers to methods that protect neurons from cerebral IRI by altering behavioral patterns or applying behavioral stimuli to influence the activity of NMDAR or its downstream signaling pathways.^[106–108]^ For example, cognitive training is an approach that utilizes a variety of cognitive tasks to improve cognitive abilities and functions. Cognitive training can improve cognitive deficits after cerebral IRI by enhancing synaptic plasticity and learning memory, thereby protecting neurons from cerebral IRI.^[3]^ Exercise training is a method of utilizing various forms of exercise to improve physical health and function. Exercise training can improve neurological function after cerebral IRI by increasing blood flow, promoting neovascularization, inhibiting inflammatory responses, and increasing neurotrophic factors, thereby protecting neurons from cerebral IRI.^[109]^

## 5. Challenges and limitations

Although NMDAR has been shown to play an important role in myocardial and cerebral IRI, there are still challenges and limitations in studying and applying NMDAR-mediated cardiac and cerebral injuries. Here, we will briefly discuss some of the major challenges and limitations in this field.

One challenge is the species differences and translational barriers between animal models and human studies. Animal models of cardioencephalic IRI are useful tools for studying mechanisms of glutamate receptor-mediated myocardial injury and therapeutic strategies, but they may not fully recapitulate the complexity and heterogeneity of human disease. For example, there are differences in the expression, distribution, and function of glutamate receptor subtypes in rodents and humans, in addition to differences in genetic background, environmental factors, comorbidities, and medications between animal models and human patients.^[9]^

Another challenge is the temporal and spatial heterogeneity of NMDAR signaling in IRI. NMDAR signaling is dynamic and context-dependent, meaning that its effects can vary based on factors such as the timing of activation, duration, location, and cell type. For example, activation of NMDAR can have both deleterious and beneficial effects on IRI, depending on the timing of activation.^[35,72]^

The third challenge is the crosstalk and redundancy of NMDAR subtypes in IRI. The NMDAR subtypes are not isolated entities but interact with each other and with other signaling pathways to regulate IRI. For example, NMDA receptor activation can affect AMPA receptor trafficking and function through calcium-dependent mechanisms,^[34]^ and we believe that the different NMDAR subunits act by the same different mechanisms during IRI,^[45]^ as well as the presence of glutamate metabolism in the circulatory system and the reuptake of NMDAR available in the brain and blood.

## 6. Prospects and future directions

NMDAR plays a key role in the pathophysiology of IRI, and modulating NMDAR offers a promising strategy for preventing and treating ischemic heart-brain injury. We believe that the following ideas and future directions can be further explored in the future: A reliable and sensitive biomarker for identifying NMDAR activation in IRI can be used for early diagnosis, prognosis, or therapeutic evaluation of IRI. It can also reflect the dynamic changes of NMDAR signaling at different stages of IRI. Additionally, elucidating the molecular mechanism of NMDAR-mediated injury in IRI from various levels of complexity, such as gene expression, protein interactions, signaling, cellular functions, network activities, and behavioral outcomes, is crucial. Furthermore, investigating the relationship between NMDAR and other factors or pathways involved in IRI is important.

## 7. Conclusions

NMDAR plays an important role in myocardial and cerebral IRI. Different NMDAR subunits have different mechanisms of action in IRI. Blocking or modulating NMDAR-specific NMDAR subunits could be an effective strategy for protecting against myocardial and cerebral IRI. Studying the role of NMDAR in IRI, understanding the regulation of upstream and downstream of NMDAR subunits, and improving the specificity of glutamate receptor inhibitors are important for further investigating NMDAR-related pathways in cardiac and cerebral injury. This research can provide new targets for clinical intervention in the treatment of cardiac and cerebral ischemia-hypoxia-reperfusion injury.

## Acknowledgments

In this study, we would like to thank all the colleagues who took part in the study.

## Author contributions

**Formal analysis:** Wei Liao, Ziyou Liu.

**Funding acquisition:** Wei Liao, Ziyou Liu.

**Methodology:** Wei Liao, Yuehui Wen, Yanyu Duan.

**Writing – original draft:** Wei Liao, Shaochun Yang, Ziyou Liu.

**Writing – review & editing:** Wei Liao, Ziyou Liu.
